# Antepartum fetal monitoring in pregnant women with diabetes: A systematic review

**DOI:** 10.1002/pmf2.70123

**Published:** 2025-09-29

**Authors:** Aoife M. Hurley, Saima Sultana, Joshua P. Vogel, Maureen Makama

**Affiliations:** ^1^ Women's, Children's and Adolescents’ Health Program Burnet Institute Melbourne Australia; ^2^ Melbourne School of Population and Global Health The University of Melbourne Melbourne Australia

**Keywords:** antepartum care, diabetes, fetal monitoring, gestational diabetes, pregnancy, systematic review

## Abstract

Diabetes in pregnancy affects approximately 15.5% of pregnant women worldwide, the majority of these women are in low‐ and middle‐income countries. Current guidelines recommend additional fetal monitoring for pregnant women with diabetes, particularly during the third trimester, though there is limited evidence supporting these recommendations. Our objective was to identify the optimal methods and regimens for antepartum fetal monitoring for pregnant women with diabetes to inform recommendations for future clinical practice. CENTRAL, Embase, MEDLINE, and LILACs databases were searched through 23 July 2024. There were no restrictions on language or publication type. We included randomized controlled trials (RCTs) or nonrandomized studies of interventions (NRSIs) involving pregnant women with diabetes (whether gestational, type 1, or type 2). Eligible studies were those investigating fetal monitoring in these women, such as fetal growth monitoring, fetal well‐being monitoring, or Doppler ultrasonography. Our comparisons of interest were (1) monitoring versus no monitoring/usual care, (2) monitoring versus a different type of monitoring, and (3) different regimens of the same type of monitoring. Meta‐analysis was not possible due to heterogeneity of the included studies; narrative synthesis was conducted and the Synthesis Without Meta‐analysis (SWiM) guidelines were followed. The GRADE approach (Grading of Recommendations, Assessment, Development, and Evaluations) was used to assess the certainty of evidence for each outcome. Five studies with 4440 pregnant women with diabetes were included; two were RCTs (239 women) and three were NRSIs (4201 women). A single ultrasound growth scan over no ultrasound growth scan probably increases the risk of caesarean section (risk ratio [RR] 1.71, 95% confidence interval [CI] 1.46–2.01, 1 study, *n* = 2357, *moderate certainty*). There is a paucity of data regarding optimal antepartum fetal monitoring for pregnant women with diabetes. Robust, high‐quality studies are urgently needed to inform clinical guidelines.

## 1 INTRODUCTION

Diabetes in pregnancy, whether pregestational or gestational, has wide‐ranging consequences for both the mother and the fetus, including increased rates of stillbirth and neonatal death [1]. Globally, the prevalence of diabetes in pregnancy was estimated to be 15.5% in 2019 [2, 3]. The majority of these cases, more than 90%, are estimated to occur in low‐ and middle‐income countries (LMICs) [3].

Antepartum fetal surveillance or monitoring techniques are used to evaluate fetal well‐being, alongside routine ultrasonography and umbilical artery Doppler velocimetry [4, 5]. The information obtained from antepartum fetal monitoring can identify potential uteroplacental compromise and thus inform obstetric care, including timing of birth. This can help reduce the risk of perinatal mortality, morbidity, and other adverse outcomes. Zamani et al.’s review of current fetoplacental monitoring practices highlighted the limitations of current available methods and the urgent need for innovation in fetal monitoring tools and technologies [6].

There are numerous guidelines on fetal monitoring for diabetes in pregnancy, which largely recommend additional antepartum fetal monitoring, particularly during the third trimester [7, 8, 9, 10]. A 2024 narrative review by Braverman‐Poyastro et al. highlighted the variation across such guidelines internationally, in terms of the recommended frequency and type of fetal monitoring [11]. However, a challenge common across these guidelines is the lack of direct evidence underpinning their recommendations. It is currently unclear what monitoring technique and regimen are optimal for pregnant women with diabetes, in terms of effectiveness, acceptability, feasibility, and cost‐effectiveness. Frequent fetal monitoring is also time‐ and resource‐intensive—this may be feasible in well‐resourced settings, but not in limited‐resource settings. More frequent antepartum monitoring might also detract from the provision of other, beneficial interventions [12].

## 2 OBJECTIVE

This review aimed to help resolve these uncertainties by evaluating the relative benefits and harms of different approaches to fetal monitoring in pregnant women with diabetes. The findings can help inform evidence‐based recommendations for clinical practice, including forthcoming World Health Organization (WHO) recommendations on diabetes in pregnancy [13].

## 3 METHODS

The review protocol was prospectively registered in PROSPERO (Prospective Register of Systematic Reviews; CRD42024557435). The findings were reported following the Preferred Reporting Items for Systematic Reviews and Meta‐Analyses (PRISMA) guidelines and the Synthesis Without Meta‐analysis (SWiM) reporting guidelines [14, 15].

### 3.1 Eligibility criteria, information sources, and search strategy

Published randomized controlled trials (RCTs) (individual parallel trials, cluster trials, and crossover trials) or nonrandomized studies of interventions (NRSIs) were eligible for inclusion. We defined NRSI according to the *Cochrane Handbook* as any quantitative, comparative study estimating the effectiveness of an intervention (harm or benefit) that does not use randomization to allocate units (whether individuals or clusters) to intervention groups [16]. Such studies include those in which allocation occurs in the course of usual treatment decisions or according to peoples’ choices (often called observational studies).

Eligible studies were those that included pregnant women with diabetes (whether gestational, type 1, or type 2) and investigated fetal monitoring for this indication. The comparisons of interest were:

1. monitoring versus no monitoring or usual care,
2. monitoring versus a different type of monitoring, and
3. different regimen of the same type of monitoring.

We used a broad, inclusive approach to antepartum monitoring interventions, though we expected to find studies on monitoring fetal growth (such as serial symphysis‐fundal height measurements [SFH] or ultrasounds) and fetal well‐being (such as nonstress test [NST] and contraction stress tests [CST], biophysical profile [BPP] or modified BPP, use of Doppler ultrasonography) and other, similar interventions.

Studies that assessed accuracy measures (such as sensitivity, specificity, positive and negative predictive values) as their primary aim were excluded unless they also reported the health effects of the antepartum fetal monitoring interventions assessed. Studies were excluded if the comparison group was chosen from a different population to the treatment group (e.g., if the comparison group were healthy individuals and the treatment group were women with diabetes). All other study designs, as well as protocols of ongoing trials, were excluded.

The search strategy was developed in collaboration with information specialists from the Cochrane Response Team. It combined terms for diabetes in pregnancy, fetal monitoring, RCTs and NRSIs (search strategies for all databases are in Appendix S1). Three databases were searched from inception until 23 July 2024 (CENTRAL, Embase, and MEDLINE) and one database (LILACs) was searched until 16 October 2024. There were no limitations in terms of date or language of publication. Google Translate was used to screen studies in a language other than English.

### 3.2 Study selection

Endnote X9 and Covidence software were used to manage citations. Two reviewers (A.M.H. and S.S.) independently assessed all studies for eligibility, at both the title abstract stage and the full‐text screening stage. Authors were contacted to request full text of potentially relevant studies that were not otherwise available. Any disagreements were resolved through discussion or consultation with a third reviewer (M.M.). Articles published in languages other than English were screened using Google Translate as a translation tool. Eligible RCTs were assessed using the Research Integrity Assessment tool [17]. This tool provides a transparent mechanism for identifying problematic trials and avoiding misleading findings. The primary outcome of interest was stillbirth/fetal death. Additional outcomes of interest were fetal growth restriction, macrosomia, birth injury, shoulder dystocia, neonatal hypoglycemia, mode of birth, and induction of labor. The complete list of secondary review outcomes is listed in Appendix S2.

### 3.3 Data extraction

Data were extracted using a customized data extraction template in Covidence [18]. Two reviewers (A.M.H. and S.S.) extracted data from each report independently, with any disagreements resolved through discussion or consultation with a third reviewer (M.M.). Extracted data included author, year of publication, country, study design, population characteristics, sample size, gestational age at enrolment, type and frequency of monitoring and review outcomes. Data were analyzed using RevMan Version 8.17.0 [19].

### 3.4 Assessment of risk of bias

Risk of bias was assessed using the revised Cochrane Risk of Bias 2 tool (RoB 2) for RCTs and the Risk Of Bias In Nonrandomized Studies of Interventions tool (ROBINS‐I) for the NRSIs [20]. Two reviewers (A.M.H. and S.S.) independently assessed the risk of bias using these tools, with disagreements resolved through discussion or consultation with another reviewer (M.M.).

### 3.5 Data synthesis

Study findings were summarized separately by intervention type. The rationale for this grouping is the expectation that fetal monitoring of different types or intensities will help identify fetuses at risk of complications and inform subsequent management, timing, and mode of birth. We planned to use random‐effects models for meta‐analysis because we anticipated substantial between‐study heterogeneity [21]. However, we found considerable dissimilarities in the type of interventions used, outcomes, and study designs, which precluded meta‐analysis. Findings were synthesized narratively following the SWiM guidelines (Appendix S3). Risk ratios were used to estimate intervention effects. No additional methods were used to transform the intervention effects from those reported in the included studies [15]. Forest plots were prepared to aid visual interpretation of the data and convey overall patterns (Appendix S5) [22]. The GRADE approach (Grading of Recommendations, Assessment, Development, and Evaluations) was used to assess the certainty of evidence for each outcome (Appendix S4) [23].

## 4 RESULTS

### 4.1 Study characteristics

**FIGURE 1:**
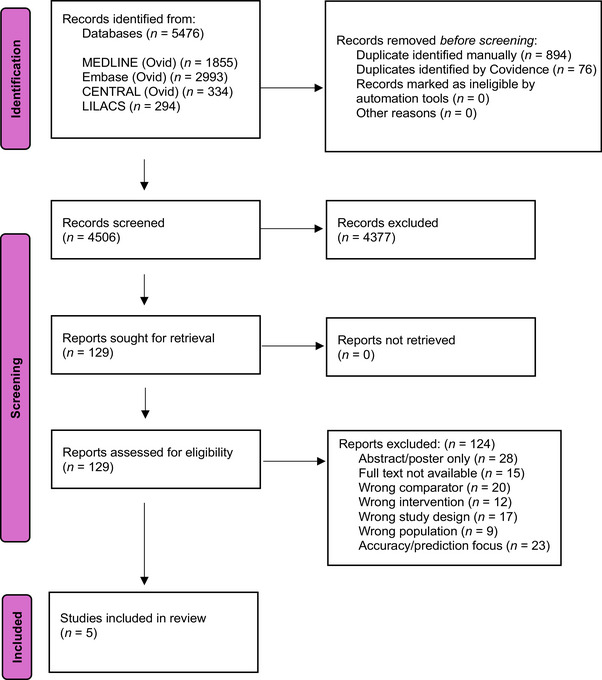
PRISMA flow diagram representing number of studies identified during the search.

The database searches identified 5476 reports. After removing duplicates, we screened 4506 titles/abstracts and 129 full texts. Of these, five studies were eligible (Figure 1). Two were RCTs [24, 25] and three were NRSIs [26, 27, 28]. The characteristics of included studies are summarized in Table 1. Studies were published between 1996 and 2017, and there were data from 4440 pregnant women with diabetes [24, 25, 26, 27, 28]; sample sizes ranged from 85 [24] to 3690 [26]. All were conducted in high‐income countries—four in the United States [24, 26, 27, 28, 29] and one in Italy [25]. In two studies, women with diabetes were a subgroup of a larger study population of women at increased risk of complications [24, 26].

**TABLE 1:** Summary of characteristics of included studies.

Study (country)	Study design	Study period	Population	Sample size	Intervention	Comparator	Outcomes	Notes
Bracero et al. 1999 [24] (United States)	RCT	Not described	Pregnant women with an indication for antepartum FHR Subgroup of women with diabetes (not otherwise specified)	Overall *n* = 410 (205 intervention, 205 comparator) Pregnant women with diabetes (not otherwise specified) *n* = 85 (38 intervention, 47 comparator)	Visual interpretation of NST GA not otherwise specified	Computerized interpretation of NST GA not otherwise specified	Perinatal morbidity*	For subgroup of women with diabetes, only one outcome of interest (perinatal morbidity) was reported
Rossi et al. 2000 [25] (Italy)	RCT	September 1994–January 1998	Pregnant women GDM Singleton	Pregnant women with GDM *n* = 154 (73 intervention, 68 comparator)	US growth scan at 28 weeks and 32 weeks	US growth scan at 32 weeks only	Mode of delivery GA at birth Macrosomia Apgar score < 7 at 5 min Neonatal hypocalcemia Hyperbilirubinemia Neonatal hypoglycemia	
Froehlich et al. 2016 [26] (United States)	C(R)	2008–2011	Pregnant women at or beyond 37 weeks of gestation Subgroup of women with diabetes (pregestational or GDM)	Overall *n* = 66,4030 (6068 intervention, 23,939 comparator 1, 34,023 comparator 2) Pregnant women with diabetes (pregestational or GDM) *n* = 3690 (616 intervention, 1333 comparator 1, 1741 comparator 2)	US EFW within 1 week of delivery	Clinical EFW within 1 week of delivery EFW not collected (undocumented)	Mode of delivery: CS	For subgroup of women with diabetes, only one outcome of interest (mode of delivery: CS) was reported
Dude et al. 2017 [27] (United States)	C(R)	1 January 2010–31 December 2015	Nulliparous women with pregestational diabetes or GDM requiring medication	Pregnant women with diabetes (pregestational or GDM requiring medication) *n* = 304 (231 intervention, 73 comparator)	US growth scan for EFW within 5 weeks (35 days) of delivery	No US growth scan for EFW within 5 weeks (35 days of delivery)	Mode of delivery: CS Induction of labor GA at birth Birthweight Birthweight by category (LGA/AGA/SGA)	
Bracero et al. 1996 [28] (United States)	C(R)	11 July 1989–11 April 1993	Pregnant women with diabetes Singleton	Pregnant women with diabetes (not otherwise specified) *n* = 207	These three tests performed concurrently within 1 week of delivery: NST BPP Doppler velocimetry (umbilical artery US)	NST nonreactive BPP ≤ 6 Umbilical artery S/D ≥ 3 S/D≥ 2.5	Mode of delivery: CS Fetal growth restriction Preterm birth (<32 weeks; <37 weeks) Hyperbilirubinemia Neonatal hypocalcemia Neonatal hypoglycemia Respiratory distress syndrome	No IRB approval or informed consent

Abbreviation: AGA, appropriate birthweight for gestational age; BPP, biophysical profile; C, Cohort study; C(R), retrospective cohort; CS, caesarean section; EFW, estimated fetal weight; FHR, fetal heart rate monitoring; GA, gestational age; GDM, gestational diabetes mellitus; LGA, large for gestational age; MA, meta‐analysis for standardized effect sizes; NEE, no effect estimate; NS, narrative synthesis; NST, nonstress test; RCT, randomized controlled trial; S/D, standard deviation; SGA, small for gestational age; US, ultrasound.

* Perinatal morbidity: defined as caesarean delivery for fetal distress, hypocalcemia (total serum calcium level 12 mg/dL), hypoglycemia (glucose serum level < 30 mg/dL), respiratory distress syndrome, transient tachypnoea and meconium aspiration.

The type of fetal monitoring varied between studies—three used ultrasound growth scans to estimate fetal weight [25, 26, 27], the fourth was a three‐arm study [28] using ultrasound monitoring for BPP, Doppler velocimetry (umbilical artery ultrasound), and NST; and the fifth used NST only. Frequency and timing of monitoring also varied—one compared ultrasound growth scans at 28 and 32 weeks to a single ultrasound growth scan at 32 weeks only [25]. Two studies examined ultrasound growth scans prior to birth—Dude et al. [27] within 5 weeks (35 days) of birth, and Froehlich et al. [26] within 1 week of birth. Two studies examined NST [24, 28]. Bracero et al. [24] compared visual interpretation of NST to computerized interpretation, though the gestational age at monitoring was not stated. Bracero et al. [28] examined NST used concurrently with BPP and Doppler velocimetry (umbilical artery ultrasound). These three tests were all performed within 1 week of birth.

### 4.2 Risk of bias and research integrity assessment

**FIGURE 2:**
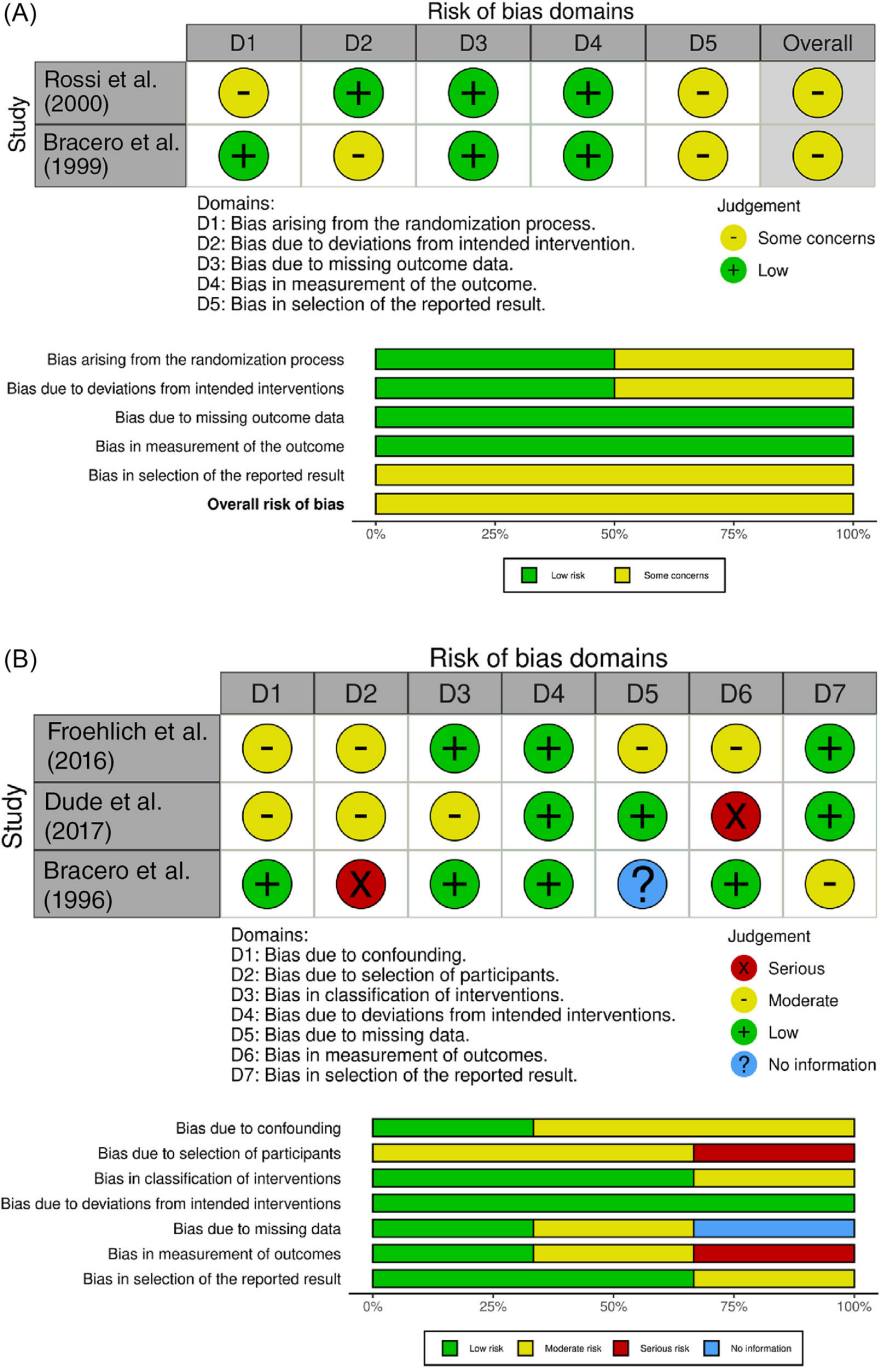
(A) Risk of bias assessment for randomized controlled trials using RoB‐2. (B) Risk of bias assessment for nonrandomized studies of interventions using ROBINS‐I.

Both RCTs [24, 25] had “some concerns” identified through risk of bias assessment. In both cases, this was due to the selection of reported results and the absence of a published analysis plan. Two NRSIs had a “serious concern” and the third NRSI had a “moderate” concern on ROBINS‐I assessment for risk of bias [26, 27, 28]. This was related to the selection of participants across the three NRSIs due to the retrospective study design [26, 27, 28], and concern that selection into the study may have been related to intervention and outcome (Figure 2). Neither of the two RCTs had been retracted or flagged for integrity concerns, and none were excluded following our research integrity assessment [17]. In one RCT, there was a concern regarding insufficient details of the randomization process; however, the authors did not respond to our emailed requests for further details [25]. A consensus decision (A.M.H., M.M., and J.P.V.) was made not to exclude the study.

### 4.3 Synthesis of results

Four comparisons, based on the intervention type, are described below. These are: (1) different regimens of ultrasound growth scan, (2) different methods of interpretating NST, (3) ultrasound growth scan versus no ultrasound growth scan, and (4) umbilical artery Doppler velocimetry versus NST versus BPP. The findings from comparisons 1–3 are summarized in the Summary of Findings tables (Appendix S4).

#### 4.3.1 Comparison 1: Ultrasound scan at 28 and 32 weeks versus ultrasound scan at 32 weeks only

Rossi et al. [25] compared ultrasound growth scans at 28 and 32 weeks versus a single ultrasound growth scan at 32 weeks only. For this comparison, there may be little to no difference in the risk of vaginal birth, though the evidence was low certainty (risk ratio [RR] 1.00, 95% confidence interval [CI] 0.83–1.21, 1 study, *n* = 141) (Appendix S5). The study provided data on several other outcomes, including Apgar score < 7 at 5 min, gestational age at birth, macrosomia, neonatal hyperbilirubinemia, hypocalcemia and hypoglycemia, instrumental birth, and caesarean section. There may be little to no difference in effect for any of these outcomes; however, the data for all these outcomes were of very low certainty, so the true effect is uncertain (Appendices S4 and S5).

#### 4.3.2 Comparison 2: Computerized interpretation versus visual interpretation of NST

For this comparison, Bracero et al. [24] reported data for stillbirth, and for a composite perinatal morbidity outcome (defined as any one or more of: caesarean for fetal distress, hypocalcemia, hypoglycemia, respiratory distress syndrome, transient tachypnoea, or meconium aspiration). There may be little to no difference in effect for either outcome; however, the data were of very low certainty (Appendices S4 and S5).

#### 4.3.3 Comparison 3: Ultrasound growth scan versus no ultrasound growth scan

Two NRSIs explored this comparison. Froehlich et al. [26] was a three‐arm study, comparing ultrasound growth scan to estimate fetal weight versus clinical estimation of fetal weight (EFW) versus no documented fetal weight (i.e., no recorded ultrasound growth scan or clinical estimation). Dude et al. [27] compared a single ultrasound growth scan to estimate fetal weight versus no ultrasound growth scan.

Froehlich et al. [26] reported only one outcome (caesarean section) for the subgroup of women with diabetes (*N* = 3690). For ultrasound EFW compared to no documented fetal weight, the risk of caesarean section was probably higher in the intervention group (RR 1.71, 95% CI 1.46–2.01, 1 study, *n* = 2357, *moderate certainty*) (Appendix S4). For ultrasound EFW compared to no ultrasound EFW (including clinical EFW and no ultrasound EFW), ultrasound growth scan is probably associated with an increase in likelihood of caesarean section (RR 1.61, 95% CI 1.40–1.86, 1 study, *n* = 3690, *moderate certainty*).

Dude et al. [27] also examined ultrasound growth scan for EFW versus no ultrasound growth scan for EFW, but within a broader timeframe of 5 weeks of delivery. Although these studies [26, 27] exhibited many similarities, the difference in timing of the intervention precluded the use of meta‐analysis. The risk of caesarean section was probably higher in the intervention group (RR 1.88, 95% CI 1.27–2.79, 1 study, *n* = 304, *low certainty*) (Appendix S5). There is probably a reduced risk of small‐for‐gestational‐age with an ultrasound growth scan (RR 0.40, 95% CI 0.21–0.75, 1 study, *n* = 304, *moderate certainty*) (Appendix S5). Birthweight and induction of labor may be higher with ultrasound growth scan (mean difference 0.2 kg higher, 0.05–0.35 and RR 1.77, 95% CI 1.33–2.36, respectively) (*n* = 304, *low certainty*) (Appendix S5). There may be little to no differences in gestational age at birth (*low certainty*), appropriate‐for‐gestational‐age birthweight (*low certainty*), or large‐for‐gestational‐age (*very low certainty*) between the groups (Appendix S5).

#### 4.3.4 Comparison 4: Umbilical artery Doppler velocimetry versus NST versus BPP

Bracero et al. compared umbilical artery Doppler velocimetry with NST and BPP to determine which test was best at predicting adverse outcomes (defined in the study as delivery before 37 weeks of gestation or the occurrence of fetal growth restriction, hypocalcemia, hypoglycemia, hyperbilirubinemia, respiratory distress syndrome, or fetal risk requiring caesarean delivery) [28]. The authors reported that in pregnant women with a nonreactive NST, adverse outcomes were higher (RR 1.7, 95% CI 1.2–2.5, 1 study, *n* = 207). For pregnant women where BPP < 6, the risk of adverse outcomes was 1.7 (RR 1.7, 95% CI 0.9–2.9, 1 study, *n* = 207). For pregnancies in which the umbilical artery systolic to diastolic ratio was ∼3.0, the relative risk of adverse outcome was 2.6 (RR 2.6, 95% CI 1.9–3.5, 1 study, *n* = 207).

## 5 DISCUSSION

### 5.1 Principal findings

This is the first systematic review to examine different fetal monitoring strategies for women with diabetes in pregnancy. Despite the ubiquitous use of additional fetal monitoring for pregnant women with diabetes, we found limited evidence to indicate whether this monitoring is beneficial, or what mode and frequency of fetal monitoring is optimal.

### 5.2 Comparison with existing literature

A 2024 narrative review by Braverman‐Poyastro et al. [11] reported on existing evidence relating to antepartum fetal surveillance in women with diabetes and identified eight international guidelines on this topic [9, 30, 31, 32, 33, 34, 35, 36]. Consistent with our findings, the authors noted the scarcity of clinical trials, which has led to a reliance on observational studies to support recommendations. Current guidance tends to extrapolate from the general obstetric population to this specific high‐risk group and often relies on consensus and expert opinion. The 2016 WHO recommendations on antenatal care for a positive pregnancy experience [37] do not recommend routine antenatal cardiotocography for the general obstetric population. A 2024 review by da Silva et al. [38] examining the impact of prenatal care on perinatal outcomes of pregnant women with diabetes found it reduced the risk of complications through early intervention. However, the scope of the review focused on prenatal care and nutritional therapy during pregnancy and did not extend to antepartum fetal monitoring. An existing Cochrane review by Alfirevic et al. on the use of fetal and umbilical Doppler ultrasound in women with high‐risk pregnancies concluded that umbilical artery Doppler in high‐risk pregnancies at risk of placental insufficiency improves perinatal outcomes, although the optimum timing and frequency of monitoring remains unclear. However, the role of additional monitoring for pregnant women with diabetes is debatable—this is consistent with our review findings.

### 5.3 Strengths and limitations

All included studies were assessed for research integrity to ensure retracted or problematic studies were not included. To minimize bias, we only included studies in which the treatment and control groups were from the same population. In general, there was significant heterogeneity in the type of interventions used, outcomes reported, and study designs, which precluded pooling data for several outcomes. The variation in risk profiles for stillbirth and other adverse perinatal outcomes between women with pregestational diabetes and gestational diabetes is well described [39]. However, the paucity of data for each group meant it was not feasible to conduct individual analyses for the separate groups. One study dominated the results as 82% of the review's population was from a single study [26]. Additionally, in two studies [24, 26] women with diabetes were a subgroup of the overall study population. All included studies were conducted in high‐income countries (four in the United States), and hence the results may not generalize to other, dissimilar settings.

### 5.4 Implications for practice and future research

The available evidence is not sufficient to draw conclusions or provide recommendations for fetal monitoring to reduce stillbirth in pregnant women with diabetes. Though the evidence is not conclusive, different approaches to fetal monitoring probably do have an effect on health outcomes. For example, caesarean section and induction use are possibly higher when an ultrasound growth scan is used, though the risk of small‐for‐gestational age might be lower. Perinatal mortality might be greater when NSTs are interpreted visually, rather than via computerized interpretation. However, it is important to note that the lack of data for fetal monitoring in this population does not mean there is no benefit. Without high‐quality studies (ideally randomized trials), variations in clinical practice and guidelines will likely persist. Low‐value care, defined as an intervention that confers little or no benefit on patients or may even cause harm, represents up to 30% of the costs of healthcare [40] and contributes to the avoidable environmental impacts and carbon emissions of healthcare delivery [41]. Identifying and reducing low‐value care and the associated economic, social, and environmental costs is an important step to meeting the urgent needs for a resilient, equitable healthcare system [42].

The lack of contemporary research reflecting current technological advances in monitoring was apparent. That is, included studies do not reflect antenatal monitoring interventions that are currently available, for example, modern cardiotocography devices that incorporate computerized analysis with machine learning algorithms [43, 44], wireless monitoring systems that facilitate remote, out‐of‐hospital monitoring [45], or improvements in Doppler ultrasound technology that provide better insights into fetal hemodynamics. New studies that reflect current care are required—these should focus specifically on diabetes in pregnancy, and report on key health outcomes, maternal functioning and well‐being, women's views and experiences of additional monitoring, as well as health system outcomes. The challenges of conducting randomized trials in this population are acknowledged, particularly when fetal monitoring techniques have become ingrained in routine antepartum care in the context of the known association with stillbirth. However, until further high‐quality trials are available, current clinical practice remains without supportive evidence to determine what (if any) fetal monitoring regimen is optimal.

## 6 CONCLUSION

This systematic review evaluated the relative benefits and harms of different antepartum fetal monitoring approaches for pregnant women with diabetes. Finding data from two RCTs and three NRSIs, we conclude that different approaches to fetal monitoring will likely affect health outcomes for women and babies. However, there is insufficient evidence to draw conclusions on which option is best—robust, high‐quality studies are urgently needed to inform clinical guidelines. Clear, evidence‐based guidance on fetal monitoring regimens that can optimize maternal and perinatal outcomes, while balancing the burden on resources, will remain out of reach until further primary evidence is generated.

## AUTHOR CONTRIBUTIONS

Aoife M. Hurley, Maureen Makama, and Joshua P. Vogel conceived the study and designed the protocol. Aoife M. Hurley, Maureen Makama, and Joshua P. Vogel developed the search strategy in collaboration with the Cochrane Response Team. Aoife M. Hurley, Saima Sultana, and Maureen Makama selected the studies and extracted relevant information. Aoife M. Hurley and Saima Sultana synthesized the data. Aoife M. Hurley wrote the first draft of the paper. All authors contributed to the interpretation of the findings, critically revised the manuscript for intellectual content and approved the final manuscript.

## CONFLICT OF INTEREST STATEMENT

The authors declare no conflicts of interest.

## Supporting information



Supporting information

Supporting information

Supporting information

Supporting information

Supporting information

## Data Availability

The data that support the findings of this study are available from the corresponding author upon reasonable request.
