# Supplementary material for: Antepartum fetal monitoring in pregnant women with diabetes: A systematic review
Source: Pregnancy (Hoboken). 2025 Sep 29;1(6):e70123. doi: 10.1002/pmf2.70123 (PMC13344709; doi:10.1002/pmf2.70123)
Supplement: Supplementary file 2 [file PMF2-1-e70123-s002.docx]

**S2 Review Outcomes**

**Standard fetal and neonatal outcomes were considered:** stillbirth/fetal death, neonatal death, perinatal death, admission to neonatal intensive care, gestational age at birth, preterm birth (<32 weeks; <37 weeks), birthweight, fetal growth restriction, low birthweight (<2500 g), small‐for‐gestational age (<10th centile for gestational age), Apgar score of <7 at 5minutes, neurodevelopmental outcomes (into infancy and childhood). **In addition to the following fetal and neonatal outcomes:** Congenital anomalies, macrosomia, large for gestational age, shoulder dystocia, birth injury, hyperbilirubinemia, respiratory distress syndrome, transient tachypnoea of the newborn, neonatal hypoglycaemia, neonatal seizures amniotic fluid abnormalities (polyhydramnios and oligohydramnios)

**Standard maternal outcomes were considered:** maternal death, antenatal hospitalisation, admission to intensive care, mode of birth, maternal functioning/well-being, women's views and experiences. **In addition to the following:** Early pregnancy loss (spontaneous or elective), hypertensive disorders in pregnancy, pre-eclampsia, induction of labour, childbirth-related injuries (i.e. perineal injuries, etc)

**Health system outcomes were considered:** antenatal admissions to hospital for the mother and length of stay, emergency department visits for the mother, length of postnatal hospitalisation for the mother, length of neonatal hospitalisation for the infant, costs of care for the mother or baby or both.
