# Supplementary material for: Antepartum fetal monitoring in pregnant women with diabetes: A systematic review
Source: Pregnancy (Hoboken). 2025 Sep 29;1(6):e70123. doi: 10.1002/pmf2.70123 (PMC13344709; doi:10.1002/pmf2.70123)
Supplement: Supplementary file 3 [file PMF2-1-e70123-s003.docx]

**Diabetes and Fetal Monitoring – Search Strategies**

**3 Ovid Multidbs (MEDLINE + Embase + CENTRAL);** July 23, 2024:

| 1. | diabetes, gestational/ or pregnancy in diabetics/ |
| --- | --- |
| 2. | (diabet* adj10 (pregnan* or gestation* or obstetric* or prenatal* or antenatal* or antepartum or perinatal* or pre-natal* or ante-natal* or ante-partum or peri-natal*)).ti,kf,hw. |
| 3. | (diabet* adj10 (eclamp* or preeclamp* or pre eclamp* or amniocentes* or chorion* vill* or cesarean or caesarean or cesarian or caesarian or cesarien or caesarien or tocoly* or fetal or foetal or fetus or foetus or miscarriage*)).ti,kf,hw. |
| 4. | or/1-3 |
| 5. | diabet*.ti,kf,hw. |
| 6. | limit 5 to pregnancy - focussed |
| 7. | diabetes mellitus/ or exp diabetes complications/ or exp diabetes mellitus, type 1/ or exp diabetes mellitus, type 2/ |
| 8. | exp Pregnancy/ or Obstetrics/ or Fetus/ |
| 9. | 7 and 8 |
| 10. | 4 or 6 or 9 |
| 11. | exp *Diabetes Insipidus/ |
| 12. | insipidus.ti,kf,hw. |
| 13. | 11 or 12 |
| 14. | 10 not 13 |
| 15. | exp fetal monitoring/ or exp ultrasonography, prenatal/ or cardiotocography/ or exp Fetal Heart/ or Ultrasonography/ or exp Ultrasonography, Doppler/ |
| 16. | (((f?etal or f?etus) adj10 (monitor* or surveillance or assess* or heart* or cardio* or cardiac*)) or ultrasonogra* or ultrasound* or ultrasonic* or sonogra* or echogra* or cardiotocogra* or cardio-tocogra*).ti,ab,kf,hw. |
| 17. | 15 or 16 |
| 18. | 14 and 17 |
| 19. | randomized controlled trial.pt. |
| 20. | controlled clinical trial.pt. |
| 21. | randomized.ab. |
| 22. | placebo.ab. |
| 23. | drug therapy.fs. |
| 24. | randomly.ab. |
| 25. | trial.ab. |
| 26. | groups.ab. |
| 27. | or/19-26 |
| 28. | exp animals/ not humans.sh. |
| 29. | 27 not 28 |
| 30. | 18 and 29 |
| 31. | exp cohort studies/ or exp epidemiologic studies/ or exp clinical trial/ or exp evaluation studies as topic/ or exp statistics as topic/ |
| 32. | ((time and factors) or program or survey* or ci or cohort or comparative stud* or evaluation studies or follow-up*).mp. |
| 33. | (control and (group* or study)).mp. |
| 34. | or/31-33 |
| 35. | (animals/ not humans/) or comment/ or editorial/ or exp review/ or meta analysis/ or consensus/ or exp guideline/ |
| 36. | hi.fs. or case report.mp. |
| 37. | or/35-36 |
| 38. | 34 not 37 |
| 39. | 18 and 38 |
| 40. | limit 18 to "systematic review" |
| 41. | (guideline or guidance or systematic review* or umbrella review or overview of reviews).ti. |
| 42. | 18 and 41 |
| 43. | "systematic review".pt. or "Systematic Reviews as Topic"/ or ("Cochrane Database of Sys-tematic Reviews" or evidence report technology assessment or evidence report technology assessment summary).jn. |
| 44. | 18 and 43 |
| 45. | 30 or 39 or 40 or 42 or 44 |
| 46. | 45 use medall |
| 47. | 4 or 9 |
| 48. | 47 not 13 |
| 49. | 17 and 48 |
| 50. | 49 use cctr |
| 51. | (diabet* adj10 (pregnan* or gestation* or obstetric* or prenatal* or antenatal* or antepartum or perinatal* or pre-natal* or ante-natal* or ante-partum or peri-natal*)).ti,kf,hw. |
| 52. | (diabet* adj10 (eclamp* or preeclamp* or pre eclamp* or amniocentes* or chorion* vill* or cesarean or caesarean or cesarian or caesarian or cesarien or caesarien or tocoly* or fetal or foetal or fetus or foetus or miscarriage*)).ti,kf,hw. |
| 53. | insipidus.ti,kf,hw. |
| 54. | (((f?etal or f?etus) adj10 (monitor* or surveillance or assess* or heart* or cardio* or cardiac*)) or ultrasonogra* or ultrasound* or ultrasonic* or sonogra* or echogra* or cardiotocogra* or cardio-tocogra*).ti,kf,hw. |
| 55. | exp maternal diabetes mellitus/ or exp gestational diabetes/ or exp pregnancy in diabetics/ |
| 56. | 51 or 52 or 55 |
| 57. | exp diabetes insipidus/ |
| 58. | 53 or 57 |
| 59. | 56 not 58 |
| 60. | exp fetus monitoring/ or cardiotocography/ |
| 61. | exp fetus echography/ |
| 62. | 54 or 60 or 61 |
| 63. | 59 and 62 |
| 64. | randomized controlled trial/ or controlled clinical trial/ or randomization/ or exp intermethod comparison/ or double-blind procedure/ |
| 65. | human experiment/ |
| 66. | (random$ or crossover or cross over or volunteer or volunteers or placebo or allocated or assigned or (open adj label)).ti,ab. |
| 67. | (compare or compared or comparison).ti. |
| 68. | ((evaluated or evaluate or evaluating or assessed or assess) and (compare or compared or comparing or comparison)).ab. |
| 69. | ((double or single or doubly or singly) adj (blind or blinded or blindly)).ti,ab. |
| 70. | parallel group$1.ti,ab. |
| 71. | ((assign$ or match or matched or allocation) adj5 (alternate or group$1 or intervention$1 or patient$1 or subject$1 or participant$1)).ti,ab. |
| 72. | (controlled adj7 (study or design or trial)).ti,ab. |
| 73. | trial.ti. |
| 74. | or/64-73 |
| 75. | (Systematic review not (trial or study)).ti. |
| 76. | (random cluster adj3 sampl$).ti,ab. |
| 77. | (review.ab. and review.pt.) not trial.ti. |
| 78. | (databases adj4 searched).ab. |
| 79. | "we searched".ab. and (review.ti. or review.pt.) |
| 80. | "update review".ab. |
| 81. | Random field$.ti,ab. |
| 82. | (nonrandom$ not random$).ti,ab. |
| 83. | (random$ adj sampl$ adj7 (cross section$ or questionnaire$1 or survey$ or database$1)).ti,ab. not (comparative study/ or controlled study/ or randomi?ed controlled.ti,ab. or randomly assigned.ti,ab.) |
| 84. | Cross-sectional study/ not (randomized controlled trial/ or controlled clinical trial/ or controlled study/ or randomi?ed controlled.ti,ab. or control group$1.ti,ab.) |
| 85. | (((case adj control$) and random$) not randomi?ed controlled).ti,ab. |
| 86. | (rat or rats or mouse or mice or swine or porcine or murine or sheep or lambs or pigs or piglets or rabbit or rabbits or cat or cats or dog or dogs or cattle or bovine or monkey or monkeys or trout or marmoset$1).ti. and animal experiment/ |
| 87. | Animal experiment/ not (human experiment/ or human/) |
| 88. | ("cochrane database of systematic reviews" or "cochrane database of systematic reviews online").jn. |
| 89. | or/75-88 |
| 90. | 74 not 89 |
| 91. | 63 and 90 |
| 92. | case control study/ or Controlled study/ or Major clinical study/ or cohort.ti. |
| 93. | 86 or 87 |
| 94. | 92 not 93 |
| 95. | 63 and 94 |
| 96. | 91 or 95 |
| 97. | 96 use emczd |
| 98. | 46 or 50 or 97 |
| 99. | remove duplicates from 98 |

**LILACS** (https://lilacs.bvsalud.org); October 16, 2024:

(fetal OR foetal OR fetus OR foetus OR feto OR embaraz* OR gravid* OR pregnan* OR gestation* OR obstetric* OR prenatal* OR antenatal* OR antepartum OR perinatal* OR pre-natal* OR ante-natal* OR ante-partum OR peri-natal* OR partum OR natal* OR eclamp* OR preeclamp* OR pre eclamp* OR pre-eclamp* OR amniocentes* OR chorion* vill* OR cesarean OR caesarean OR cesarian OR caesarian OR cesarien OR caesarien OR newborn* OR new born* OR tocoly* OR miscarriage* OR birth* OR childbirth* OR stillbirth* OR intrauterine OR uterine OR abortion* OR apgar OR congenital OR macrosomia OR dystocia) AND (monitor* OR ultrasonogra* OR ultrasound* OR ultrasoni* OR sonogra* OR echogra* OR cardiotocogra* OR cardio-tocogra*) AND (diabet*) AND db:("LILACS") AND type_of_study:("observational_studies" OR "clinical_trials" OR "evaluation_studies" OR "health_technology_assessment" OR "systematic_reviews") AND (instance:"lilacsplus")
