# Supplementary material for: Antepartum fetal monitoring in pregnant women with diabetes: A systematic review
Source: Pregnancy (Hoboken). 2025 Sep 29;1(6):e70123. doi: 10.1002/pmf2.70123 (PMC13344709; doi:10.1002/pmf2.70123)
Supplement: Supplementary file 4 [file PMF2-1-e70123-s004.docx]

| **Summary of findings: Comparison 1** | | | | | | |
| --- | --- | --- | --- | --- | --- | --- |
| **Ultrasound scan at 28 and 32 weeks compared to ultrasound scan at 32 weeks only for fetal monitoring in women with diabetes** | | | | | | |
| **Patient or population:** fetal monitoring in women with diabetes  **Setting:** Italy  **Intervention:** Ultrasound scan at 28 and 32 weeks  **Comparison:** ultrasound scan at 32 weeks only | | | | | | |
| Outcomes | **Anticipated absolute effects^*^** (95% CI) | | Relative effect  (95% CI) | № of participants  (studies) | Certainty of the evidence  (GRADE) | Comments |
|  | **Risk with ultrasound scan at 32 weeks only** | **Risk with Ultrasound scan at 28 and 32 weeks** |  |  |  |  |
| Vaginal birth | 750 per 1,000 | **750 per 1,000**  (622 to 908) | **RR 1.00**  (0.83 to 1.21) | 141  (1 RCT) | ⨁⨁◯◯  Low^a,b^ |  |
| Instrumental (ventouse/forceps) birth | 0 per 1,000 | **0 per 1,000**  (0 to 0) | **RR 2.80**  (0.12 to 67.52) | 141  (1 RCT) | ⨁◯◯◯  Very low^a,c,d^ |  |
| Caesarean section | 250 per 1,000 | **233 per 1,000**  (130 to 418) | **RR 0.93**  (0.52 to 1.67) | 141  (1 RCT) | ⨁◯◯◯  Very low^a,b,d^ |  |
| Gestational age at birth | The mean gestational age at birth was **0** | MD **0.13 lower**  (0.58 lower to 0.32 higher) | - | 141  (1 RCT) | ⨁⨁◯◯  Low^a,b^ |  |
| Macrosomia | 176 per 1,000 | **109 per 1,000**  (48 to 252) | **RR 0.62**  (0.27 to 1.43) | 141  (1 RCT) | ⨁◯◯◯  Very low^a,c,d^ |  |
| Neonatal hypoglycaemia | 147 per 1,000 | **137 per 1,000**  (60 to 309) | **RR 0.93**  (0.41 to 2.10) | 141  (1 RCT) | ⨁◯◯◯  Very low^a,c,d^ |  |
| Hyperbilirubinemia | 103 per 1,000 | **96 per 1,000**  (35 to 259) | **RR 0.93**  (0.34 to 2.52) | 141  (1 RCT) | ⨁◯◯◯  Very low^a,c,d^ |  |
| Neonatal hypocalcaemia | 88 per 1,000 | **82 per 1,000**  (28 to 243) | **RR 0.93**  (0.32 to 2.75) | 141  (1 RCT) | ⨁◯◯◯  Very low^a,c,d^ |  |
| Apgar score <7 at 5 minutes | 44 per 1,000 | **41 per 1,000**  (8 to 197) | **RR 0.93**  (0.19 to 4.46) | 141  (1 RCT) | ⨁◯◯◯  Very low^a,c,d^ |  |
| ***The risk in the intervention group** (and its 95% confidence interval) is based on the assumed risk in the comparison group and the **relative effect** of the intervention (and its 95% CI).   **CI:** confidence interval; **MD:** mean difference; **RR:** risk ratio | | | | | | |
| **GRADE Working Group grades of evidence**  **High certainty:** we are very confident that the true effect lies close to that of the estimate of the effect.  **Moderate certainty:** we are moderately confident in the effect estimate: the true effect is likely to be close to the estimate of the effect, but there is a possibility that it is substantially different.  **Low certainty:** our confidence in the effect estimate is limited: the true effect may be substantially different from the estimate of the effect.  **Very low certainty:** we have very little confidence in the effect estimate: the true effect is likely to be substantially different from the estimate of effect. | | | | | | |

#### Explanations

a. Some concerns about risk of bias for the outcome

b. Estimate based on small sample size

c. Estimate based on small sample size and few events

d. Wide confidence interval including appreciable harm and appreciable benefit

| **Summary of findings: Comparison 2** | | | | | | |
| --- | --- | --- | --- | --- | --- | --- |
| **Computerised compared to visual interpretation of non-stress test** | | | | | | |
| **Population:** Pregnant women with diabetes  **Setting:** United States of America  **Intervention:** Computerised interpretation of non-stress test  **Comparison:** Visual interpretation of non-stress test | | | | | | |
| Outcomes | **Anticipated absolute effects^*^** (95% CI) | | Relative effect (95% CI) | № of participants (studies) | Certainty of the evidence (GRADE) | Comments |
|  | **Risk with visual** | **Risk with Computerised** |  |  |  |  |
| Perinatal morbidity | 263 per 1,000 | **171 per 1,000** (74 to 389) | **RR 0.65** (0.28 to 1.48) | 85 (1 RCT) | ⨁◯◯◯ Very low^a,b,c^ |  |
| Stillbirth | 0 per 1,000 | **0 per 1,000** (0 to 0) | **RR 0.27** (0.01 to 6.46) | 85 (1 RCT) | ⨁◯◯◯ Very low^a,b,c^ |  |
| ***The risk in the intervention group** (and its 95% confidence interval) is based on the assumed risk in the comparison group and the **relative effect** of the intervention (and its 95% CI).  **CI:** confidence interval; **RR:** risk ratio | | | | | | |
| **GRADE Working Group grades of evidence** **High certainty:** we are very confident that the true effect lies close to that of the estimate of the effect. **Moderate certainty:** we are moderately confident in the effect estimate: the true effect is likely to be close to the estimate of the effect, but there is a possibility that it is substantially different. **Low certainty:** our confidence in the effect estimate is limited: the true effect may be substantially different from the estimate of the effect. **Very low certainty:** we have very little confidence in the effect estimate: the true effect is likely to be substantially different from the estimate of effect. | | | | | | |

#### Explanations

a. Some concerns about risk of bias for the outcome

b. Wide confidence interval including appreciable harm and appreciable benefit

c. Estimate based on small sample size and few events

| **Summary of findings: Comparison 3** | | | | | | | |
| --- | --- | --- | --- | --- | --- | --- | --- |
| **Ultrasound growth scan compared to No Ultrasound growth scan for antepartum fetal monitoring in pregnant women with diabetes mellitus** | | | | | | | |
| **Patient or population: Women with diabetes**  **Setting: United States of America**  **Intervention:** Ultrasound growth scan  **Comparison:** No ultrasound growth scan | | | | | | | |
|  | | | | | | | |
| Outcomes | **Anticipated absolute effects^*^** (95% CI) | | Relative effect  (95% CI) | № of participants  (studies) | | Certainty of the evidence  (GRADE) | Comments |
|  | **Risk with No Ultrasound growth scan** | **Risk with Ultrasound growth scan** |  |  |  |  |  |
| Caesarean section | 171 per 1000 | **292 per 1000**  (249 to 343) | **RR 1.71**  (1.46 to 2.01) | 2357  (1 non-randomised study) | | ⨁⨁⨁◯  Moderate^a^ |  |
| Induction of labour | 0 per 1000 | **0 per 1000**  (0 to 0) | **RR 1.77**  (1.33 to 2.36) |  | 304  (1 non-randomised study) | ⨁⨁◯◯  Low^a^ |  |
| Appropriate birthweight gestational age | 699 per 1000 | **789 per 1000**  (671 to 936) | **RR 1.13**  (0.96 to 1.34) | 304  (1 non-randomised study) | | ⨁◯◯◯  Very low^a,b^ |  |
| Large for gestational age | 96 per 1000 | **100 per 1000**  (44 to 222) | **RR 1.04**  (0.46 to 2.32) | 304  (1 non-randomised study) | | ⨁◯◯◯  Very low^a,b^ |  |
| Small for gestational age | 205 per 1000 | **82 per 1000**  (43 to 154) | **RR 0.40**  (0.21 to 0.75) | 304  (1 non-randomised study) | | ⨁⨁◯◯  Low^a^ |  |
| Gestational age at birth (weeks) | The mean gestational age at birth (weeks) was **38.5** weeks | MD **0 weeks**  (0.26 lower to 0.26 higher) | - | 304  (1 non-randomised study) | | ⨁⨁◯◯  Low^a^ |  |
| Birthweight | The mean birthweight (Kilograms) was **3.227** Kilograms (Kg) | MD **0.2 Kilograms (Kg) higher**  (0.05 higher to 0.35 higher) | - | 304  (1 non-randomised study) | | ⨁⨁◯◯  Low^a^ |  |
| Caesarean section | **Low** | | **RR 1.88**  (1.27 to 2.79) | 304  (1 non-randomised study) | | ⨁⨁◯◯  Low^a^ |  |
|  | 0 per 1000 | **0 per 1000**  (0 to 0) |  |  |  |  |  |
| **GRADE Working Group grades of evidence**  **High certainty:** we are very confident that the true effect lies close to that of the estimate of the effect.  **Moderate certainty:** we are moderately confident in the effect estimate: the true effect is likely to be close to the estimate of the effect, but there is a possibility that it is substantially different.  **Low certainty:** our confidence in the effect estimate is limited: the true effect may be substantially different from the estimate of the effect.  **Very low certainty:** we have very little confidence in the effect estimate: the true effect is likely to be substantially different from the estimate of effect. | | | | | | | |

#### Explanations

^a^Some concerns about risk of bias for the outcome

^b^Wide confidence interval including appreciable harm and appreciable benefit
