# Supplementary material for: Antepartum fetal monitoring in pregnant women with diabetes: A systematic review
Source: Pregnancy (Hoboken). 2025 Sep 29;1(6):e70123. doi: 10.1002/pmf2.70123 (PMC13344709; doi:10.1002/pmf2.70123)
Supplement: Supplementary file 5 [file PMF2-1-e70123-s005.docx]

**Appendix S5**

**Comparison 1: Ultrasound growth scan at 28 and 32 weeks versus ultrasound growth scan at 32 weeks only**

**A**

**Vaginal birth**

**B**

**Apgar score <7 at 5 minutes**

**C**

**Gestational age at birth**

**D**

**Macrosomia**

**E**

**Neonatal hyperbilirubinemia**

**F**

**Neonatal hypocalcaemia**

**G**

**Neonatal hypoglycaemia**

**H**

**Instrumental (ventouse/forceps) birth**

**I**

**Caesarean section**

**Figure 1.** Forest plots for intervention *ultrasound growth scan at 28 and 32 weeks versus ultrasound growth scan at 32 weeks* by outcome **A)** Vaginal birth **B)** Apgar score <7 at 5 minutes **C)** Gestational age at birth **D)** Macrosomia **E)** Neonatal hyperbilirubinemia **F)** Neonatal hypocalcaemia **G)** Neonatal hypoglycaemia **H)** Instrumental (ventouse/forceps) birth **I)** Caesarean section

**A**

**Perinatal Morbidity***

******Perinatal morbidity was defined as any one or more of: caesarean for fetal distress, hypocalcaemia (total serum calcium level 12 mg/dL), hypoglycaemia (glucose serum level <30 mg/dL), respiratory distress syndrome, transient tachypnoea, and meconium aspiration. Patients with congenital anomalies were excluded from the outcome analyses.*

**B**

**Stillbirth**

**Figure 2.** Forest plot for intervention visual interpretation of cardiotocography (CTG) versus computerised interpretation of CTG **A)** Perinatal Morbidity **B)** Stillbirth

**A**

**Small-for-gestational age**

**B**

**Birthweight (Kilograms)**

**C**

**Induction of labour**

**D**

**Gestational age at birth (weeks)**

**E**

**Appropriate-for-gestational-age**

**F**

**Large-for-gestational-age**

**G**

**Caesarean Section**

**Figure 3.** Forest plots for intervention *ultrasound growth scan EFW versus no ultrasound growth scan EFW* by secondary outcome **A)** Small-for-gestational-age **B)** Birthweight (kilograms) **C)** Induction of labour **D)** Gestational age at birth (weeks) **E)** Appropriate-for-gestational-age **F)** Large-for-gestational-age **G)** Caesarean section.
